# Is It Possible to Find Needles in a Haystack? Meta-Analysis of 1000+ MS/MS Files Provided by the Russian Proteomic Consortium for Mining Missing Proteins

**DOI:** 10.3390/proteomes8020012

**Published:** 2020-05-23

**Authors:** Ekaterina Poverennaya, Olga Kiseleva, Ekaterina Ilgisonis, Svetlana Novikova, Arthur Kopylov, Yuri Ivanov, Alexei Kononikhin, Mikhail Gorshkov, Nikolay Kushlinskii, Alexander Archakov, Elena Ponomarenko

**Affiliations:** 1 Institute of Biomedical Chemistry, Moscow 119121, Russia; olly.kiseleva@gmail.com (O.K.); ilgisonis.ev@gmail.com (E.I.); novikova.s.e3101@gmail.com (S.N.); a.t.kopylov@gmail.com (A.K.); yurii.ivanov@rambler.ru (Y.I.); alexander.archakov@ibmc.msk.ru (A.A.); 2463731@gmail.com (E.P.); 2 Skolkovo Institute of Science and Technology, Skolkovo 143026, Russia; konoleha@yandex.ru; 3 V.I. Kulakov National Medical Research Center of Obstetrics, Gynecology and Perinatology, Moscow 117198, Russia; 4 V.L. Talrose Institute for Energy Problems of Chemical Physics, Federal Research Center of Chemical Physics, Russian Academy of Sciences, Moscow 119334, Russia; mike.gorshkov@gmail.com; 5 Moscow Institute of Physics and Technology (State University), Dolgoprudny 141700, Russia; 6 Blokhin Russian Cancer Research Center, Moscow 115478, Russia; kne3108@gmail.com

**Keywords:** human proteome, missing proteins, uncertain proteins, neXtProt, proteotypic peptide, mass spectrometry, Chromosome-Centric Human Proteome Project (C-HPP)

## Abstract

Despite direct or indirect efforts of the proteomic community, the fraction of blind spots on the protein map is still significant. Almost 11% of human genes encode missing proteins; the existence of which proteins is still in doubt. Apparently, proteomics has reached a stage when more attention and curiosity need to be exerted in the identification of every novel protein in order to expand the unusual types of biomaterials and/or conditions. It seems that we have exhausted the current conventional approaches to the discovery of missing proteins and may need to investigate alternatives. Here, we present an approach to deciphering missing proteins based on the use of non-standard methodological solutions and encompassing diverse MS/MS data, obtained for rare types of biological samples by members of the Russian Proteomic community in the last five years. These data were re-analyzed in a uniform manner by three search engines, which are part of the SearchGUI package. The study resulted in the identification of two missing and five uncertain proteins detected with two peptides. Moreover, 149 proteins were detected with a single proteotypic peptide. Finally, we analyzed the gene expression levels to suggest feasible targets for further validation of missing and uncertain protein observations, which will fully meet the requirements of the international consortium. The MS data are available on the ProteomeXchange platform (PXD014300).

## 1. Introduction

The chromosome-centric “Human Proteome” project (C-HPP) celebrates its 10th anniversary in 2020 (http://www.c-hpp.org/ [1]). The major goal of the project is to detect previously unreported (missing) proteins [2], since the lack of experimental evidence of gene products at the protein level casts doubt on the functional significance of the corresponding protein-coding genes. The missing fraction constitutes 10.7% of the human master proteome (where at least one protein product is detected per each protein-coding gene [3]) and includes 2129 missing proteins with transcript (PE2), homological (PE3) or prediction (PE4) statuses, and 576 uncertain proteins (PE5) support of “protein existence”, according to the neXtProt tiers (neXtProt, rel. 2019-01-11).

The speed of proteome deciphering by efforts of the International Consortium is not constant: the less proteins are missing, the more determination, ingenuity, and time is required for the detection of the next one missing protein. A more effective project realization may be required to deviate from the chromosome-centric approach and to concentrate efforts on the exploration of the hidden part of proteome, without referring to a certain chromosome.

The technical limitations of the current analytical methods constitute a tough roadblock on the way to unravelling proteomes, thus hampering the detection of low-abundant proteins [4,5]. Moreover, the detectability of such proteins is associated with the number of methodological and biological challenges [6], namely, with the conservation level of the protein sequence, availability for proteases, ionizability of proteotypic peptides, exposure to mutations and modifications, and the exploration degree and specificity of the tissue under study.

For the preliminary estimation of the protein detectability, the range of tissues where a gene is expressed is of special significance. It is natural that genes expressed in several tissues could be detected more easily than tissue-specific ones. UniGene data [7] demonstrated a good correlation between the number of experiments where peptides were detected and the number of tissues with corresponding transcripts. Experimental evidence of a protein was obtained for more than 90% of 6286 genes, expressed in 24 or more tissues. On the other hand, peptides were detected in less than one fourth of 2932 genes, with a proved expression on the transcriptomic level in two or fewer tissue types [6]. It can be assumed that genes with a broad tissue expression are more evolutionary conserved and encode proteins presented in higher concentrations, thus being more likely to be detected. Therefore, this strategy, which focuses on the re-analysis of MS data obtained for rare types of biological samples, seems to be suitable for “missing” protein searches.

Alongside the aforementioned reasons, the number of undetected proteins depends on data quality criteria. As expected, the introduction of more rigid standards [8,9,10,11,12] decreases the number of eligible experiments. The development of analytical methods inevitably leads to the expansion of the data obtained in proteomic experiments. This means that the evolution of bioinformatics methods is required to reduce false protein identifications.

In this study, we carried out a meta-analysis of the mass spectrometry data accumulated by the Russian proteomic consortium, which includes more than 1170 experiments, 2041 technical runs, and 25 types of biological samples and cell lines. The re-analysis considered the identification requirements [11] demanded by the international association for missing proteins search. Inspired by other studies [13,14], we believe that such a comprehensive re-analysis of rare MS-data will be fruitful for uncovering the dark part of the human proteome, as it was for drafting the human proteome in 2014.

## 2. Materials and Methods

### 2.1. neXtProt Data Analysis

Human protein-coding gene annotations were downloaded for each release of the neXtProt database. There are 29 releases, and the additional information providing protein evidence for each protein-coding gene started appearing in neXtProt since 2011 (six releases). Python (v. 2.7) was used for data processing: we downloaded all the versions of neXtProt, extracted lists of protein-coding genes and monitored changes in the level of evidence for corresponding proteins through the evolution of the database.

### 2.2. Expression Data Analysis

Data on gene expression (level 3 RSEM (RNA-Seq by Expectation-Maximization) normalized and log-transformed) were downloaded from the Cancer Genome Atlas portal (TCGA, http://cancergenome.nih.gov) using the RTCGA 1.14 package (https://rtcga.github.io/RTCGA). R and Python scripts were used to perform the TCGA data processing. At first, the data were log2-transformed, and values less than 0.4 were set to 0, as likely background noise (the 0.4 threshold was selected by examining distributions of log2-RSEM expression values). We limited our analysis to proteins that presented in canonical and spliced forms in the neXtProt database (neXtProt, rel. 2019-01-11). Log2-RSEM expression values of less than 0.4 were set to 0, as background noise. Transcripts expressed in less than 3/4 of the samples for each tissue (cancer and normal tissues separately) were filtered out.

### 2.3. Virtual Proteolysis

The virtual proteolysis using six proteases (Try, ArgC, AspN, GluC, LysC, and LysN) [15] was performed for human protein sequences (neXtProt, rel. 2019-01-11). We filtered out peptides that were too short or too long (out of 9–25 a.a. range) and peptides with more than one mis-cleavage. We estimated the probability of experimental peptide detection by their frequency in the global proteome repository, GPMdb [16].

### 2.4. Re-Analysis of MS Data

We encompassed diverse data obtained by mass spectrometry by members of the Russian Proteomic community on non-trivial biological materials during the last 5 years. The profile of the analyzed objects included blood plasma samples from patients with different pathologies, including oncology (colon, kidney, ovarian, breast), chorionic and liver tissues, melanoma, glioblastoma and prostate biopsies, blood cells from patients with cardiovascular diseases, intraocular and cervicovaginal fluids, placenta, urine, and HepG2, HL-60, HeLa, and Caco-2 cell lines. Raw tandem mass spectrometry (MS/MS) data were converted into an mgf format by MSConvert (v. 3.0.20130) using the “peak picking” filter [17] and processed in a uniform manner by three search engines (X!Tandem [18], MS-GF+ [19], OMSSA [20]), which are part of the SearchGUI (v. 3.3.15) package [21], coupled with PeptideShaker (v. 1.16.40) [22]. The acquired liquid chromatography coupled with mass spectrometry (LC-MS/MS) data were searched against the human neXtProt library (rel. 2019-01-11) and enriched with CRAPome contaminants [23]. The mass tolerances were set to 10 ppm and 0.5 Da for precursors and fragments, correspondingly. The carbamidomethylation of cysteine residues was set as a fixed modification, and the oxidation of methionine was allowed as a variable modification. Only highly confident peptides, according to the target-decoy approach, were accepted. A cut-off level FDR (false discovery rate) < 1% for both peptides and proteins was set. Two detected peptides (at least one of them required to be unique) were required for protein identification. For special cases we used the IdentiPROT [24], with the same parameters, for cross-validation of the detected peptides.

All MS data are available on ProteomeXchange platform (PXD014300).

## 3. Results and Discussion

### 3.1. “Protein Existence” Features for Human Protein-Coding Genes

Formally, the task of missing proteins mining is a continuation of the Human Genome Project (HGP [25]). The most important part of this is to clarify the true number of protein-coding genes in the genome. If they all encode a protein, whose existence can be reliably (according to the guidelines [12]) confirmed at the protein level?

Here, to assess changes in the rate of missing proteins detection over time we performed a comparative analysis of versions of the neXtProt platform, the main aggregator of proteomic data in the framework of the Human Proteome Project (HPP) [26].

**Figure 1:**
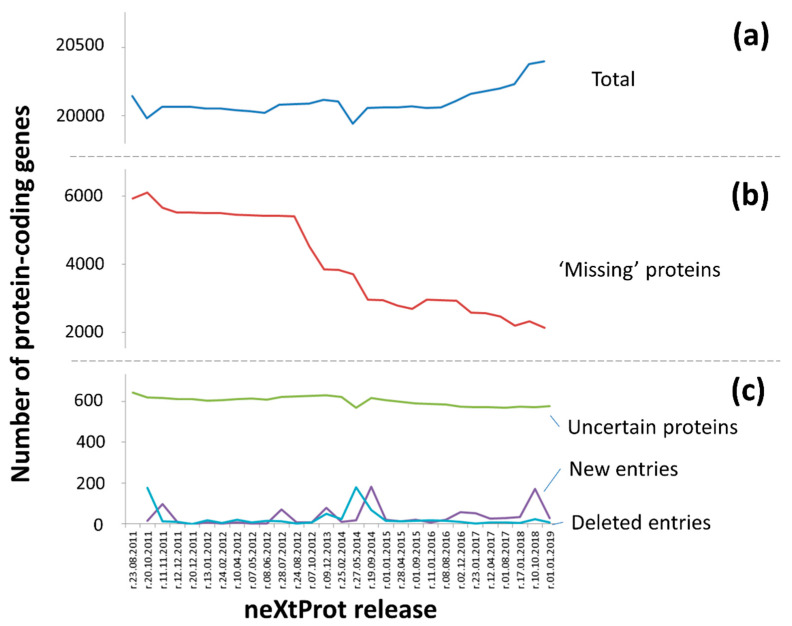
Dynamics of the changes in the number of entries according to neXtProt (2011–2019 years): (**a**) the blue color indicates the total number of entries (number of protein-coding genes); (**b**) the red color indicates the number of missing-protein entries (PE2+PE3+PE4), and (**c**) the green, purple and blue colors indicate the number of uncertain (PE5), new and deleted entries, respectively.

Figure 1 shows the dynamics of changes in the number of entries in the neXtProt from 2011 to 2019. Thus, the number of protein-coding genes (PCG) has insignificantly increased since the project started in 2008 only by 20% from 18,609 (UniProt, rel. 2008) to 20,399 in the current base version (neXtProt 01-2019, Figure 1a). About 500 entries were removed from the list of human protein-coding genes (Figure 1c), while ca. 750 were added.

Surprisingly, we obtained MS-data that confirmed the existence of proteotypic peptides belonging to proteins in entries deleted from neXtProt. It seems that these cases need to be carefully re-analyzed to exclude a hasty curator’s conclusion as well as some technical artifacts [27,28].

The total sum of PCG resulted from the number of identified proteins (PE1, existence at the protein level), missing proteins (PE2, PE3, and PE4) and dubious protein products encoded by uncertain genes (PE5). Uncharacterized genes arise from a constant adjustment and clarifications of the number of protein-coding genes in the human genome. Additionally, the number of PE5 entries remains almost constant over the analyzed period (Figure 1c).

In contrast, the number of missing proteins decreased by two thirds (Figure 1b). The main reduction was due to evidence of protein translation during the implementation of the HPP being found. There are three main experimental methods which that can be utilized to assign the PE1 status (”evidence at the protein level”): mass spectrometry, antibodies and the experimental detection of protein-protein interactions [29].

C-HPP required the strictest criteria for the mass spectrometry results, as the main method for detecting missing proteins [12], while for other methods, the guidelines were not so clearly indicated. Since the first publication in 2012, the status of PE1 has been assigned to more than 3.5 thousand of protein-coding genes (about 18% of the total number of PCG). The greatest number of reconsidered changes occurred after the acceptance of the guidelines acceptance (however, this was significantly reduced after a stringent re-analysis of the mass spectra), while the second wave came in 2014, when two drafts of the human proteome were published [13,14].

At the transcriptome level, gene products were detected on average for three-quarters of the missing protein-coding genes, with a total of 17,694 genes (neXtProt, v. 11-01-2019). In general, translation was confirmed for genes with a PE2 status (“evidence at transcript level”), while detection of the PE5 (“uncertain”) entries was extremely rare. According to neXtProt, for most cases (about 88% of PCG), the PE1 status was confirmed by mass spectrometry, while for 41 genes, there is no information about the utilized methods, and for 16 genes entries have weak evidence.

Using the antibodies, the PE1 status was assigned to four genes (TM4SF20 (Q53R12), CLDN17 (P56750), ARMS2 (P0C7Q2) and LTB4R2 (Q9NPC1)), and for the remaining entries, the PE1 status is confirmed by the results of interactomic experiments, performed mainly using the Y2H method [30]. The applicability of this method is questionable, because it involves the insertion and artificial upregulation and translation of a gene in another organism. On the one hand, the Y2H methods are known for being non-specific and producing false positive results [31]. On the other hand, the Y2H method may indicate the possibility of translation from the gene, but not in the human body.

Today, when there is a targeted manual hunt for missing proteins, the number and quality of neXtProt annotations are of great relevance, as the main backbone for evaluating the efforts of C-HPP teams.

### 3.2. Is the mRNA a Good Helper in Searching for the Missing Proteins?

An analysis of neXtProt showed that the probability of missing proteins detection is significantly higher for PE2 entries (“evidence at the transcript level”) in comparison with PE3 and PE4 entries. Thus, the presence of mRNA for the corresponding gene in the sample indicates the type of biomaterial where the missing protein can be found.

The presence of mRNA in many tissues increases the possibility of a mass spectrometric identification of the corresponding protein in comparison with proteins encoded by tissue-specific mRNA. According to UniGene [7], there is a good correlation between the number of experiments in which the peptide was detected and the number of tissues expressing the corresponding transcript [6].

To assess the tissue specificity of the mRNA corresponding to these missing and uncertain proteins, transcriptome data for 22 types of organs and tissues (cancer and normal), obtained from TCGA [32], were analyzed. On average, in each type of biomaterial, both in the normal and tumor tissues, about half of the genome is expressed (11,981 ± 304 and 11,930 ± 217, respectively), which is consistent with the results of other major projects, such as GTeX [33] or RNA-seq Atlas [34]. It is noteworthy that the total number of expressed genes formed only 65% of the total number of PCG in the human genome. The majority—9542 of 13,109 genes—are expressed in all tissue types. Thus, the expression of a substantial part of human genes is either strictly tissue-specific, or rather low. In this case, strict criteria for transcript identification (cut-off level gene expression for RNA-Seq data and Ct for PCR [4]) do not allow it to be distinguished from noise.

Among the broadly expressed genes, there are many entities from the list of missing and uncertain proteins (Table 1), with most having a PE2 status according to neXtProt. There are 274 and 58 genes specifically expressed in normal and cancer cells, respectively. Such genes are expressed in condition-specific mode. Interestingly, 161 genes are expressed in all the tissues under study, but each tissue has its own coherence (each could be a normal or tumor tissue as well). This category increases the cases in which gene A was observed in tissue a (strictly normal), tissue b (strictly cancer), tissue c (strictly normal), etc.

There are more missing and uncertain proteins among the unique transcripts for normal (98 of 274) and tumor (24 of 58) samples. In total, transcripts were found for 25% of all known PE5 genes, 24%—for PE4, 22%—for PE3 and about 55% for PE2.

The search strategy for missing and uncertain proteins based on preliminary transcriptome analysis seems very promising. The most sensitive proteomic method—selected reaction monitoring with stable isotopically labeled standards [35]—allows us to detect only a half of the proteins corresponding to the total forms of mRNA in the same sample [36].

Quantitative data on the mRNA and proteins copies are only moderately correlated [37,38]. Various biological factors, experimental artifacts and even the type of statistical analysis are the reasons for the weak correlation observed in these studies [28,39]. At the same time, transcriptome data can also provide us with preliminary information as to which of the proteoforms prevail or is solely expressed?

According to the TCGA data, for ~20% of genes the non-canonical transcript variant is expressed [40]. This information is important in the case of the search for missing proteins search via targeted MS-approaches, since the splice variant of a protein requires specific proteotypic standards. Improperly selected peptide standards can distort experimental results even if the protein is presented in the sample.

### 3.3. MS Detectable or Not?

**Figure 2:**
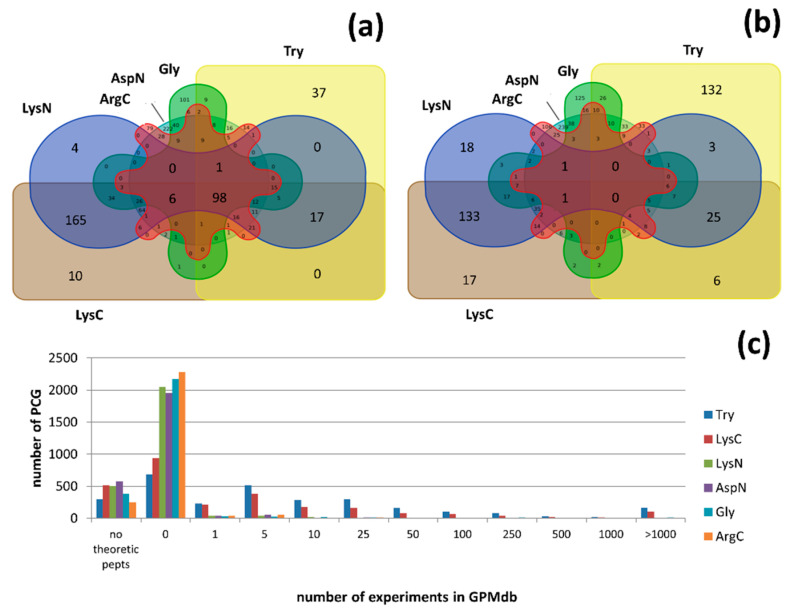
Venn diagrams: intersection of proteins cleaved by different proteases with (**a**) no proteotypic peptides at all, and (**b**) one unique peptide. (**c**) Histograms of the frequencies of the detection of proteotypic peptides, according to GPMdb. The "no peptides" group corresponds to proteins without even theoretically unique peptides, "0" means that there is no experimental evidence of theoretical proteotypic peptides, and other numbers (1, 5, 10, etc.) mean that this number of proteotypic peptides was detected in a number of cases, illustrated by the height of the corresponding column.

Genes expressed but not detected on the protein level genes naturally give rise to a question: Is it possible to detect such gene products using mass spectrometry? Choong et al. [41] demonstrated that 229 human proteins cannot be characterized with unique peptides, even if three different proteases are used, which makes their MS-detection highly questionable. In cases in which there are no standard length restrictions for proteotypic peptides, 145 proteins remain potentially undetectable, and 58 of them have a PE1 status. Setting more rigid MS-criteria, namely the peptide length, number of detected peptides and their SAP-liability, there still remain ca. 11% (300 of 2705) genes, the protein products of which are potentially undetectable through their tryptic peptides. The other five most popular proteases (ArgC, AspN, GluC, LysC, and LysN) [15] do not solve the problem completely: 323, 387, 506, 518, and 579 protein products remain MS-undetectable with ArgC, AspN, GluC, LysC, and LysN, respectively. Sequential proteolysis with multiple proteases still does not provide any proteotypic peptides for 98 gene products (Supplementary Table S1, Figure 2a).

However, single unique peptide is not sufficient for reliable protein identification, according to the basic rules of the C-HPP Consortium, except in special cases, when another unique peptide cannot be expected by any common digestion proteases [42]. Virtual proteolysis with Try provided only one unique peptide for 335 gene products, while usage of ArgC, GluC, LysN, LysC, and AspN demonstrated the same result for 253, 295, 310, 314, and 436 gene products, respectively. Supposedly, proteolysis with multiple proteases (Figure 2b) should solve this problem, because different proteases generate different peptides [43,44]. However, for 75 gene products cleaved by several proteases, there is either only one unique peptide or no proteotypic peptides at all (Supplementary Table S1). The simultaneous (not sequential) use of multiple proteases for the same aliquot of the sample will obviously decrease the probability of reliable identification due to a shortening of the resulting peptides.

In total, the identification of 149 missing and 24 uncertain proteins by MS is challenging according to in silico experiments (Supplementary Table S1). Moreover, not every theoretical peptide could be detected by the pattern of their physical-chemical properties [45]. The occurrence of peptides detected in GPMdb experiments was analyzed to evaluate the real power of proteomic mass spectrometry in the identification of proteins produced by uncertain PCGs. The main goal of this study was to estimate protein detectability, and the peptide origin (organisms, organs, and tissues under study) was therefore ignored. For 9% of missing and uncertain proteins, a proteotypic peptide occurs once only in five experiments; for other 20% of such proteins, it occurs once in 10 experiments. One third of missing and uncertain proteins do not have experimental evidence on the peptide level at all, even using trypsin, the most popular protease. Figure 2c vividly demonstrates the high degree of complexity of the detection of PE2-PE4 and especially PE5 proteins. For LysN, AspN, and GluC, the situation is more drastic: there is no MS evidence for more than 95% of missing and uncertain proteins.

The data presented in GPMdb are far from complete. The proteomic repository provides information about only 16% of the possible proteotypic peptides (22,423 of 138,326), characterizing 737 of the 2075 missing proteins. Thus, all of the accumulated information still does not provide an answer to the question regarding the “bottom up” MS efficacy in the identification of 598 missing and 139 uncertain proteins.

### 3.4. Unique Cases—beyond the C-HPP Scope

If the genes are expressed, and there are no restrictions for their detection using MS, it is natural to optimize the sample preparation procedure to detect low-copied proteins. Fractionation [46] and irreversible binding [47] are too complicated for implementation in high-throughput research. The second way is to study rare biomaterials or states of the body [48]. Both of these types of data are not commonly available in public resources and stay out the view of human proteome researchers. For missing and uncertain proteins searches, we analyzed all human proteome MS profiling experiments accumulated over the past five years by Russian scientists using mass spectrometric experiments on human proteomic profiling. The analyzed data pool included samples with a special preparation protocol (2DE, separation into fractions—the nucleus, cytoplasm, etc.), or a rare body condition (prediabetes in pregnant women, oncology, including melanoma, glioblastoma, breast cancer, etc.), or a rare type of biomaterial (umbilical cord blood, placenta, aborted material, etc.). Part of the data was previously published [49,50,51,52,53,54,55,56], and some of them were internal proteomic profiling experiments. In total, 25 types of biomaterials were analyzed, where seven proteins were detected by two peptides (one of which is unique) with varying degrees of reliability—two missing and five uncertain (Table 2, Supplementary note 1, Supplementary Table S2). Among the obtained list of proteins, we detected two missing proteins: P22532 (PE2) and A0A087WSY6 (PE3). The identification of the Immunoglobulin kappa variable 3D-15 protein (A0A087WSY6) with the one unique peptide, ASQSVSSNLAWYQQKPGQAPR (Supplementary note 1, p.S1), attracts particular attention. This peptide has been found in more than 34.5 thousand mass spectrometric experiments. The protein, A0A087WSY6, is highly similar (98% identity) to the Immunoglobulin kappa variable 3-15 protein (P01624). The difference between these two proteins is two amino acid residues (T73I and S97I), and in our results, we observed a unique peptide with a first substitution. It is also notable that the substitution of threonine with isoleucine is relatively infrequent [57].

Keratinocyte protein P22532 is compact (72 amino acid residues) and rich in prolines, which is why it has only one unique tryptic peptide (CPEPCPSPK). Another peptide for P22532, by which it can be identified, according to the recommendations of C-HPP 8–10, contains one mis-cleavage. Different variants were detected in our samples (Supplementary note 1).

Despite the fact that peptide fragmentation and mass spectra are not flawless, these peptides look very promising as targets for future SRM (Selected Reaction Monitoring) validation. The good potential of these peptides was supported with additional verification through FDR evaluation in group-specific mode using the software Scavager [58], which shows improved efficiency compared with the other popular algorithms. Scavager is based on the “target-decoy” approach. This algorithm protects analysis from overfitting by means of the creation of two control groups. The first group contains both true and false identifications, while the second group consists of false findings only (as many as false identifications in the first group). The mathematical model of Scavager is trained to distinguish between identifications from first and second groups (details described in [58]).

In our study, for one missing protein and three uncertain proteins identified with two peptides, the FDRs calculated using Scavager do not exceed 1%. The FDRs of the remaining identifications slightly exceeded 1% (<2%), which in the case of a small test sample and the special status of the target proteins is an acceptable result, practiced by the proteomic community [59,60].

Three PE5 proteins (Q58FF3, Q58FG1, Q9BYX7) were identified by unique peptides, with a good reliability and fragmentation. For the unstable protein, HSP 90-beta-3 (Q58FF7), two unique peptides were found, however, in this case, the quality of their fragmentation is doubtful, and their identification definitely requires further validation. The situation is similar with the putative nascent polypeptide-associated complex subunit alpha-like protein (Q9BZK3), for which only 5 peptides are known, where only IEDLSQEAQLAAAEK was seen in more than 2 thsd. experiments, according to GPMdb and 518 experiments, according to PeptideAtlas. The most contradictory is the putative tubulin-like protein alpha-4B (Q9H853), the sequence of which is highly similar to that of other tubulins from the family. In particular, the tryptic peptide for Q9H853 QIFHPEQLITGK cannot be considered as unique, because it contains two isoleucines and one leucine. The inability to distinguish between isoleucine and leucine using MS makes it impossible to differentiate the desired protein from the multitude of the family characterized by the peptide QLFHPEQLITGK.

### 3.5. One Hit Wonder!

Taking into account both the theoretical estimations (when even observed gene expressions do not guarantee protein MS detection, while meeting C-HPP guidelines) and re-analysis of experimental data (stressing out the uniqueness of each case in terms, for example, of the availability of one and only one proteotypic peptide), we reprocessed the obtained proteomic data with weakened restrictions regarding a single unique peptide. In total, we identified 149 proteins, among which 130 were missing (103 PE2, 19—PE3, and two PE4) and 25 uncertain (Supplementary Table S1).

**Figure 3:**
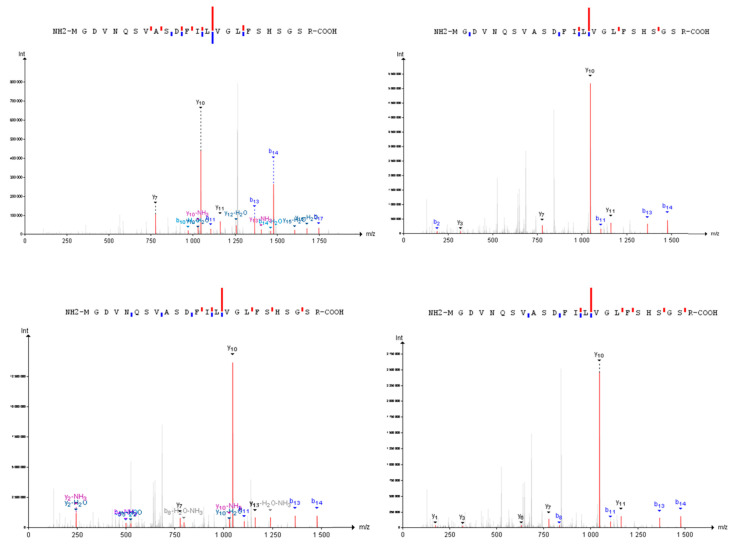
Mass-spectra of the proteotypic peptide characteristic for the Q8NG97 protein, detected in four biosamples for the first time.

The majority of the identified proteins (94%) were found in one type of biomaterial, and 104 proteins (~70%) were observed in one biological sample. Such isolated cases are presented mostly by genes with PE2 (69 PCGs) and virtually all PE5 (21 of 25) statuses. We detected 58 new peptides, never observed in GPMdb and PeptideAtlas before. However, for eleven of them, the synthetic peptides are available, according to PeptideAtlas. While the theoretical probability of detection is high (129 genes coded 110 missing and 19 uncertain proteins, with more than 10 promising peptides), the experimental evidence (not less than 10 peptides for at least in one experiment) is available only for 57 PCGs that coded 52 missing and five uncertain proteins. For the protein Olfactory receptor 2Z1 (Q8NG97) the proteotypic peptide was detected by mass spectrometry for the first time. The peptide was observed in several biological samples of melanomas (Figure 3).

For a series of missing proteins (A4D1E1 (PE2), A6NKB5 (PE2), A6NJ46 (PE2), A0AVI2 (PE2), P0C7T8 (PE2), P83859 (PE2), Q8N326 (PE2), and Q8N7P7 (PE2)) and one uncertain protein Q9UKY3 (PE5), multiple unique peptides were detected. However, only for NKX6-3 (A6NJ46) were they detected in one type of biomaterial, but in different biological samples.

**Figure 4:**
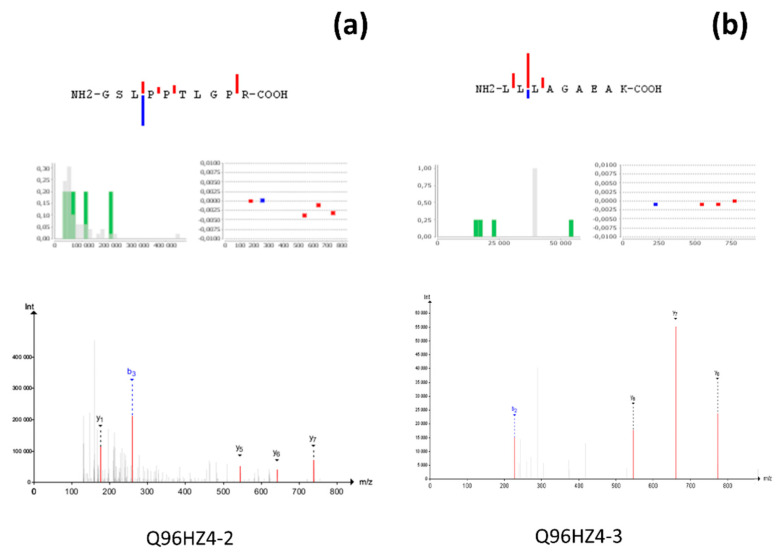
Mass-spectra of the detected proteotypic peptides for (**a**) Q96HZ4-2 and (**b**) Q96HZ4-3.

Doubtless, the majority of detected protein identifying peptides map either on a canonical form or on a group of amino acid sequences encoded by one gene (gene-specific). In accordance with the transcriptome data analysis, if there are several possible alternatively spliced transcripts, the prevailing form may either disagree with the canonical form or may be the only transcript expressed. Gene-specific peptides were detected for 32 protein-coding genes and, furthermore, for four splice forms encoded by 3 genes, DHRS12 (A0PJE2-3), LINC01547 (P58512-3), and HES6 (Q96HZ4-2, Q96HZ4-3). Several unique and MS-detectable peptides are known for these protein sequences. However, the peptide, mapping on A0PJE2-3, was registered for the first time. The prevalence of the aberrant proteoform over the canonical form is illustrated by two related types of biomaterial—liver tissue and hepatoblastoma cell line HepG2 (Figure 4). Proteome profiling for HepG2 and liver cells was performed in accordance with the same protocol, including two-dimensional gel electrophoresis [51,55]. The study revealed unique peptides for Q96HZ4-2 (one of 24 specific) and Q96HZ4-3 (one of three specific) in liver tissue and the HepG2 cell line, respectively.

## 4. Conclusions

Thanks to the tremendous efforts of the International community, about 90% of the master [10,11] proteome has been illuminated. This means that practically for each protein-coding gene, a confident experimental identification of the protein has been obtained, which reflects the status of PE1 (“evidence at the protein level”) in the neXtProt database. Now, we are challenged with 2129 genes encoding so-called missing proteins, whose detection is nontrivial and requires time-consuming approaches.

For a substantial part of these genes, there is transcript-level (mRNA) evidence in some types of biological material. Further experimental MS-based detection of the protein could be ineffective for various reasons. For example, in some cases, it is impossible to predict the proteotypic peptide due to the amino-acid features of the protein. In some cases, the physicochemical peptide properties limit detection via mass-spectrometry approaches. It seems necessary to use the alternative approaches to prove the protein existence, such as antibody-based methods or interactomic analysis. The difficulty is that specific data quality requirements must be considered in relation to these alternative approaches.

Mass spectrometric methods naturally have their own detection limit. If the biological sample contains an insufficient amount of the characteristic peptide, the expression of the corresponding protein can be modulated by gene-editing methods [61]. Another strategy implies the selection of a specific biological material, where the protein is synthesized in a high-copy mode due to the underlying biology. In this work, a re-analysis of the MS-data from more than 20 different rare types of biological material was fruitful and enabled us to confirm the evidence for seven new proteins (two missing and five uncertain) using two detected peptides and 149 new singleton proteins.

Here, in the final stage of the master human proteome analysis, it seems reasonable to move away from the chromosome-centric approach and focus on all the missing proteins. The use of non-standard methodological solutions and types of biomaterial will likely be used by future proteomics in the illumination of unknown fragments of the proteomic landscape. Taking into account this creative task, we invite the international community to reconsider specific quality requirements for the proteomic data on missing and uncertain proteins.

## Figures and Tables

**Table 1:** Distribution of the PE2, PE3, PE4, and PE5 proteins among TCGA entries.

Category of Biomaterial, Where Gene of Interest Was Observed	Total Number of Genes	Missing Proteins			Uncertain Proteins (PE5)
		PE2	PE3	PE4	
All biomaterials	9542	311	10	6	79
Part of biomaterials	3074	429	41	8	56
Normal or tumor biomaterials *	161	46	15	0	3
Only normal	274	55	36	3	4
Only cancer	58	12	8	0	4
Total	13,109	853	110	17	146

* This category included genes specifically observed in the normal or tumor states of different types of biomaterial, but not in both states of the chosen tissue.

**Table 2:** List of missing and uncertain proteins identified with two peptides.

#	AC	Gene	Number of Samples	Number of Unique Detectable Tryptic Peptides		
				Theoretically	Observed in GPMdb	Observed (SRM synt) in PeptideAtlas
Missing proteins						
1	P22532	SPRR2D	10	1	1	1/0
2	A0A087WSY6	IGKV3D-15	3	1	1	1/0
Uncertain proteins						
3	Q58FF3	HSP90B2P	1	10	3	1/2
4	Q58FG1	HSP90AA4P	1	14	13	7/5
5	Q9BYX7	POTEKP	3	8	8	5/5
6	Q9BZK3	NACA4P	1	5	5	4/4
7	Q9H853	TUBA4B	35	9	4	2/4

## Supplementary Materials

The following are available online at https://www.mdpi.com/2227-7382/8/2/12/s1, Table S1: List of undetectable proteins according to in silico proteolysis, identified as missing and uncertain proteins by one peptide. Table S2: List of MS-detected peptides mapping on missing and uncertain proteins. Supplementary Note 1: Mass-spectra of peptides, which were used for protein identification (with two peptides).
