# Supplementary material for: Is It Possible to Find Needles in a Haystack? Meta-Analysis of 1000+ MS/MS Files Provided by the Russian Proteomic Consortium for Mining Missing Proteins
Source: Proteomes. 2020 May 23;8(2):12. doi: 10.3390/proteomes8020012 (PMC7356824; doi:10.3390/proteomes8020012)

# Meta-analysis of Chr18 Consortium MS-data for missing proteins mining: is it possible to find needles in a haystack?

Ekaterina V. Poverennaya[1*], Olga I. Kiseleva[1], Ekaterina V. Ilgisonis[1], Svetlana E. Novikova[1], Arthur T. Kopylov[1], Yuri D. Ivanov[1], Alexei S. Kononikhin[2,3], Mikhail V. Gorshkov[4,5], Nikolai E. Kushlinskii[6], Alexander I. Archakov[1], Elena A. Ponomarenko[1]

1 - Institute of Biomedical Chemistry, Moscow, Russia
2 - Skolkovo Institute of Science and Technology, Skolkovo, Russia
3 - V.I. Kulakov National Medical Research Center of Obstetrics, Gynecology and Perinatology, Moscow, Russia
4 - V.L. Talrose Institute for Energy Problems of Chemical Physics, Russian Academy of Sciences, Moscow, Russia
5 - Moscow Institute of Physics and Technology (State University), Dolgoprudny, Russia
6 - "N.N. Blokhin National Medical Research Centre of Oncology" of the Health Ministry of Russia, Moscow, Russia

* k.poverennaya@gmail.com

## Supplementary Note 1.

**Mass-spectra of peptides, which were used for protein identification (with 2 peptides):**

1. A0A087WSY6 (PE3) – p. S1
2. P22532 (PE2) – p. S2
3. Q9BYX7 (PE5) – p. S3
4. Q9BZK3(PE5) – p. S4
5. Q58FF3 (PE5) – p. S5
6. Q58FG1(PE5) – p. S6
7. Q9H893(PE5) – p. S7

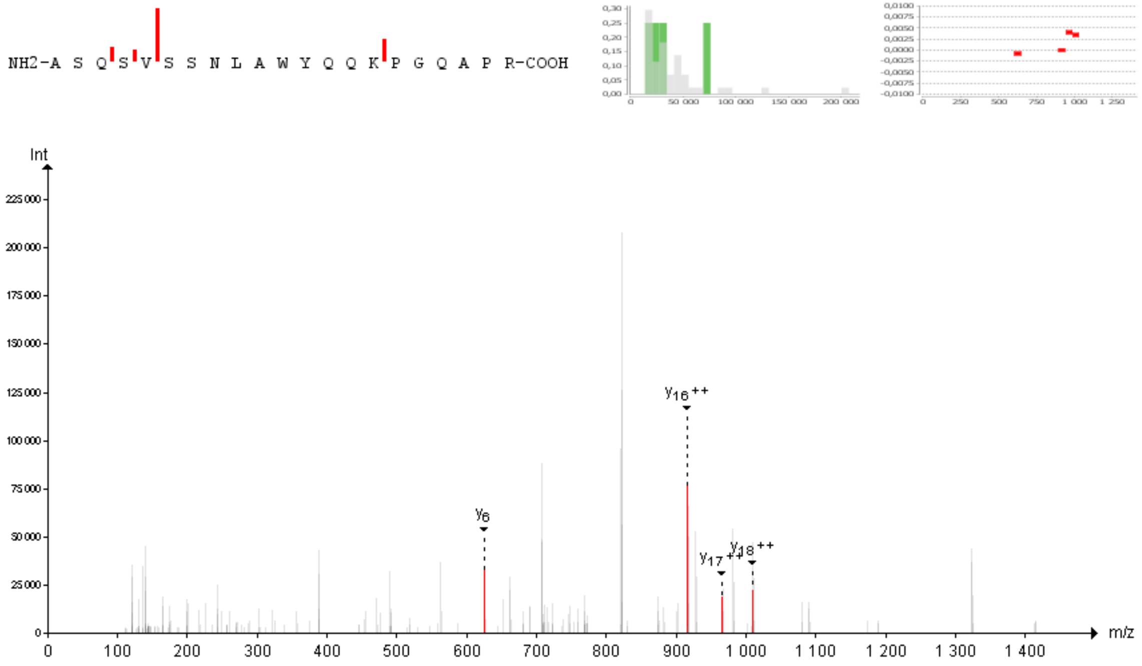


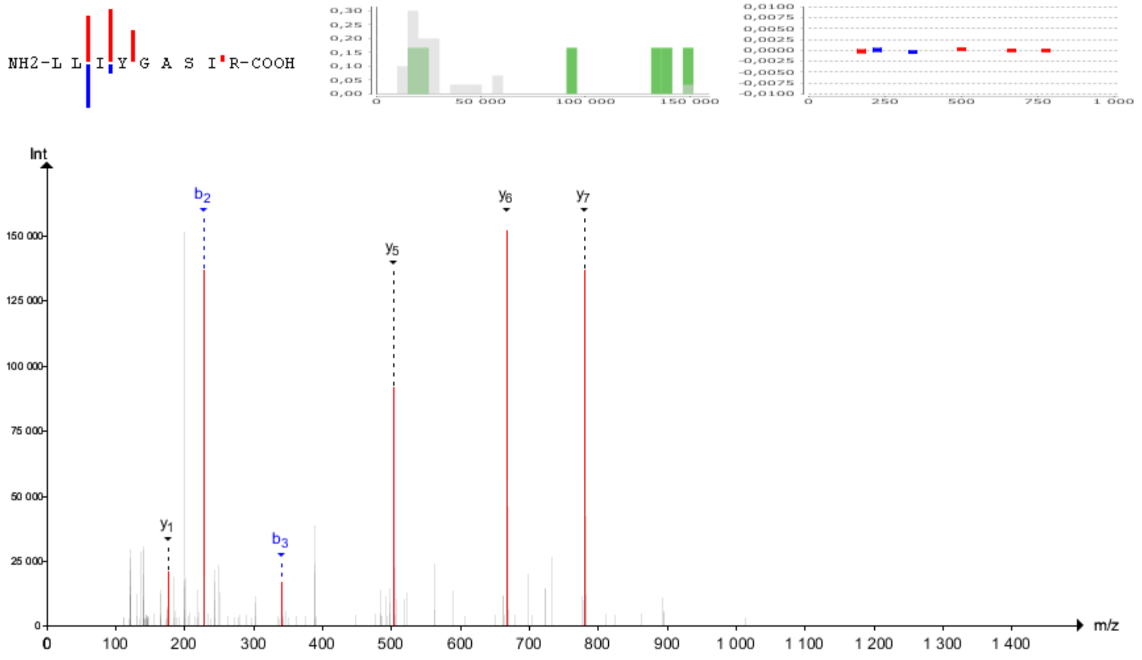


*AC:*
**A0A087WSY6**

*Protein existence:*
**PE3**

*Protein name:*
**Immunoglobulin kappa variable 3D-15**

*Gene name:*
**IGKV3D-15**

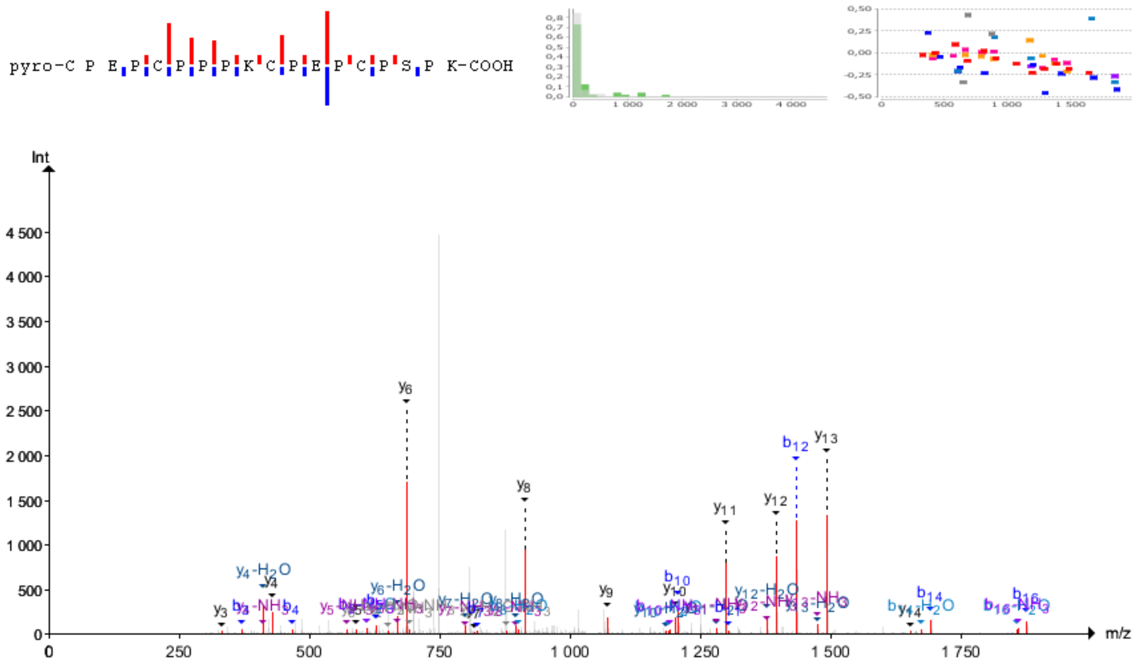


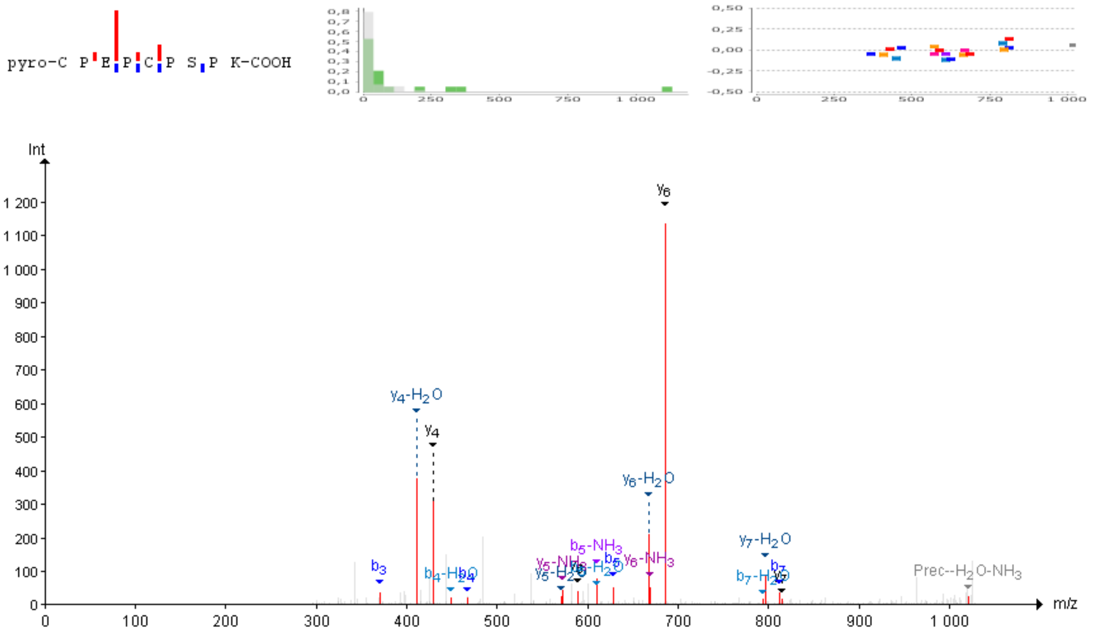


*AC:*

**P22532**

*Protein existence:*

**PE2**

*Protein name:*

**Small proline-rich protein 2D**

*Gene name:*

**SPRR2D**

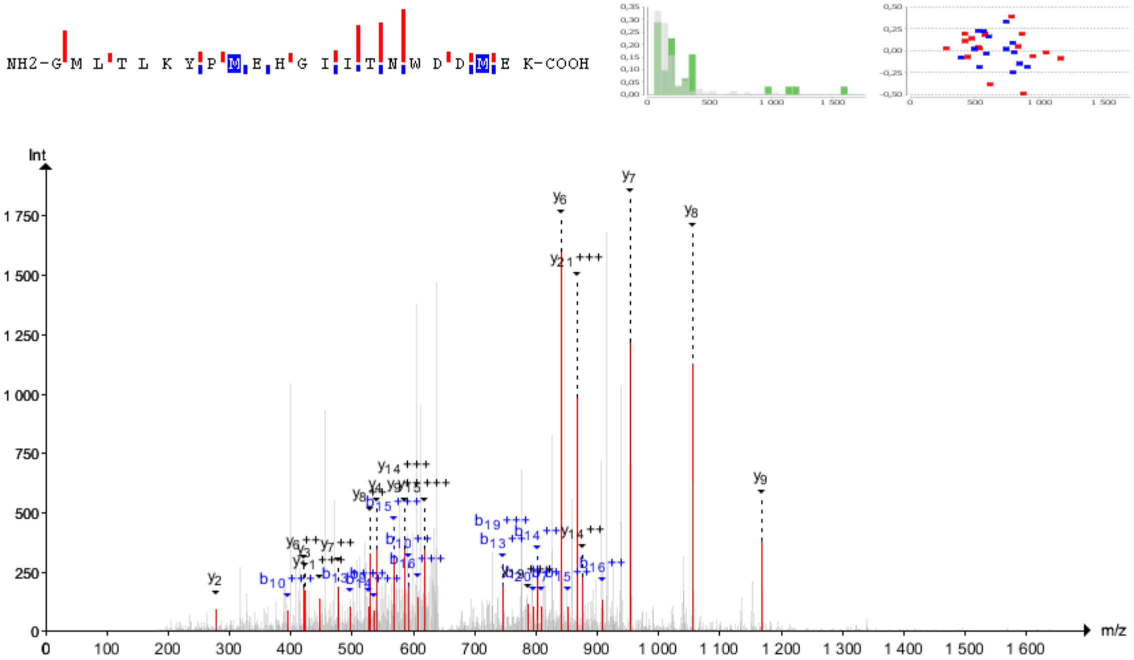


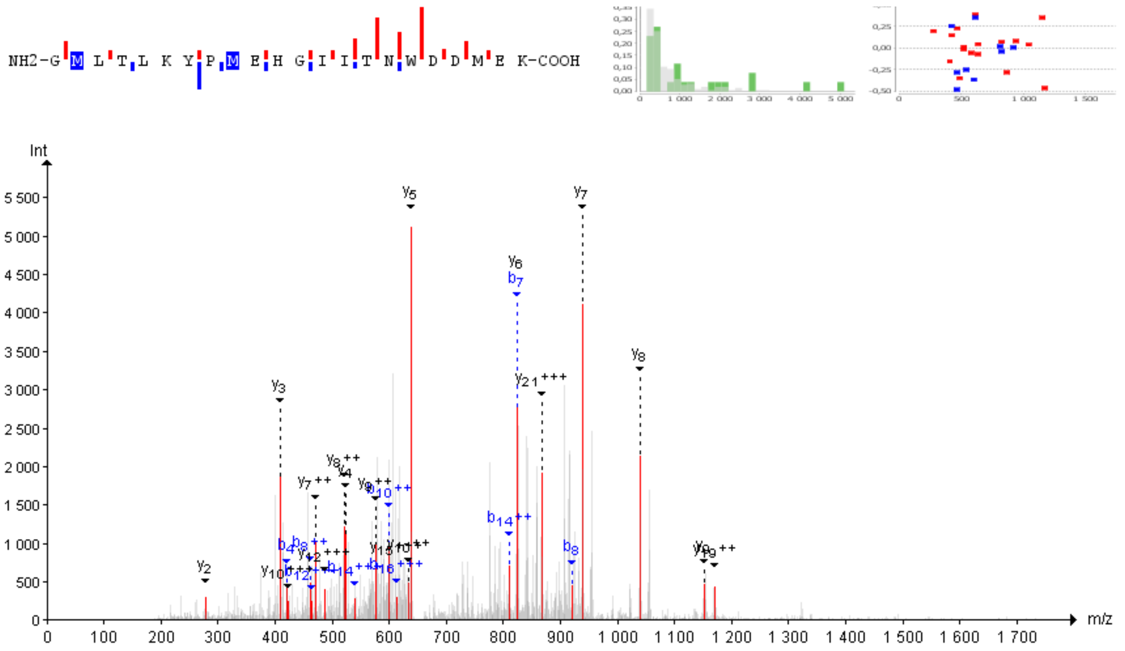


*AC:*
**Q9BYX7**

*Protein existence:*
**PE5**

*Protein name:*
**Putative beta-actin-like protein 3**

*Gene name:*
**POTEKP**

*AC:*

**Q9BZK3**

*Protein existence:*

**PE5**

*Protein name:*

**Putative nascent polypeptide- associated complex subunit alpha-like protein**

*Gene name:*

**NACA4P**

NH2-I E D L S Q E A Q L A A A E K-COOH

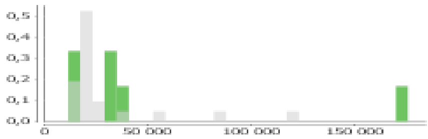


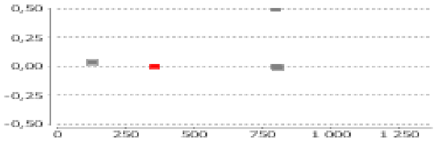


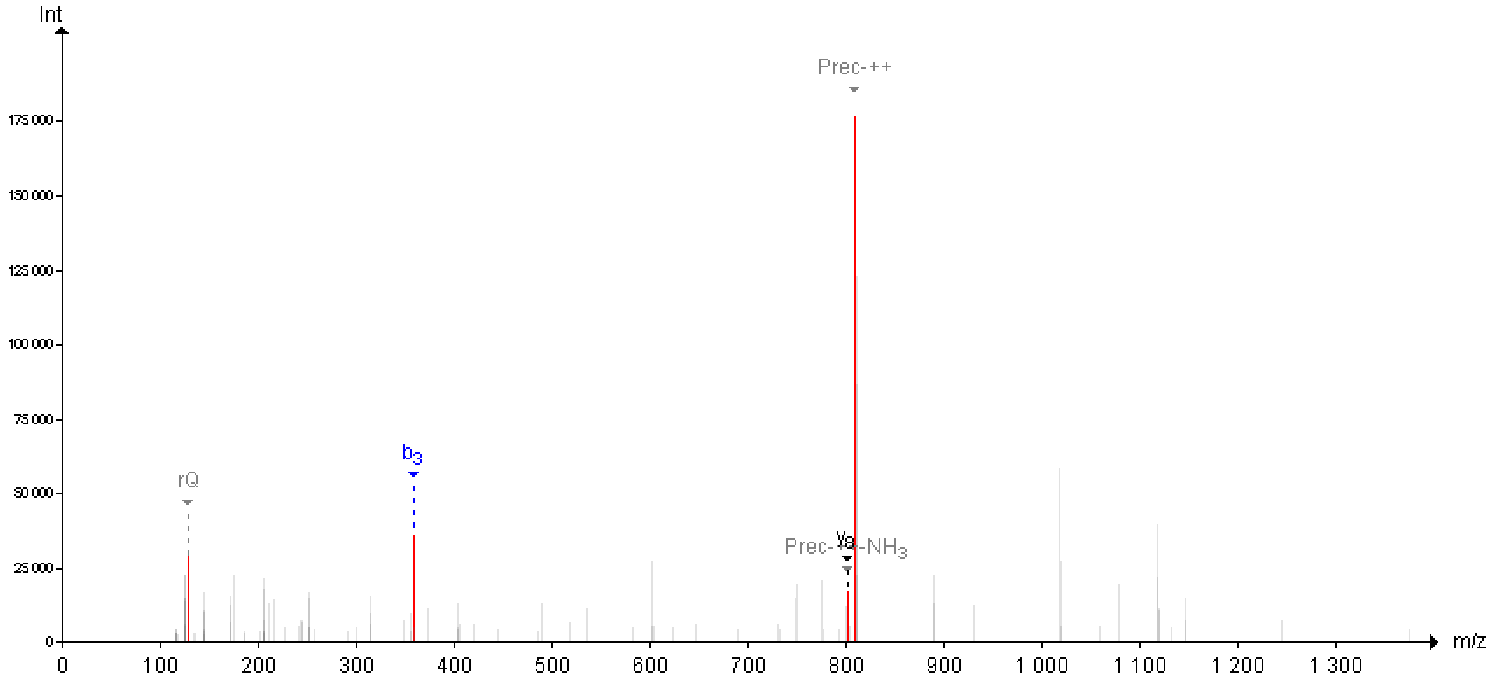

*AC:*

**Q58FF3**

*Protein existence:*

**PE5**

*Protein name:*

**Putative endoplasmin-like protein**

*Gene name:*

**NACA4P**

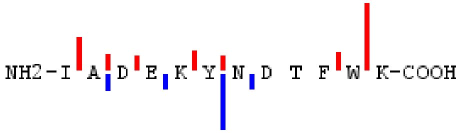


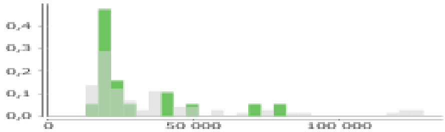


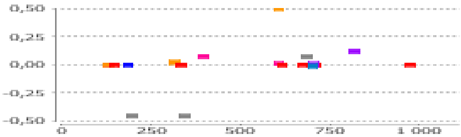


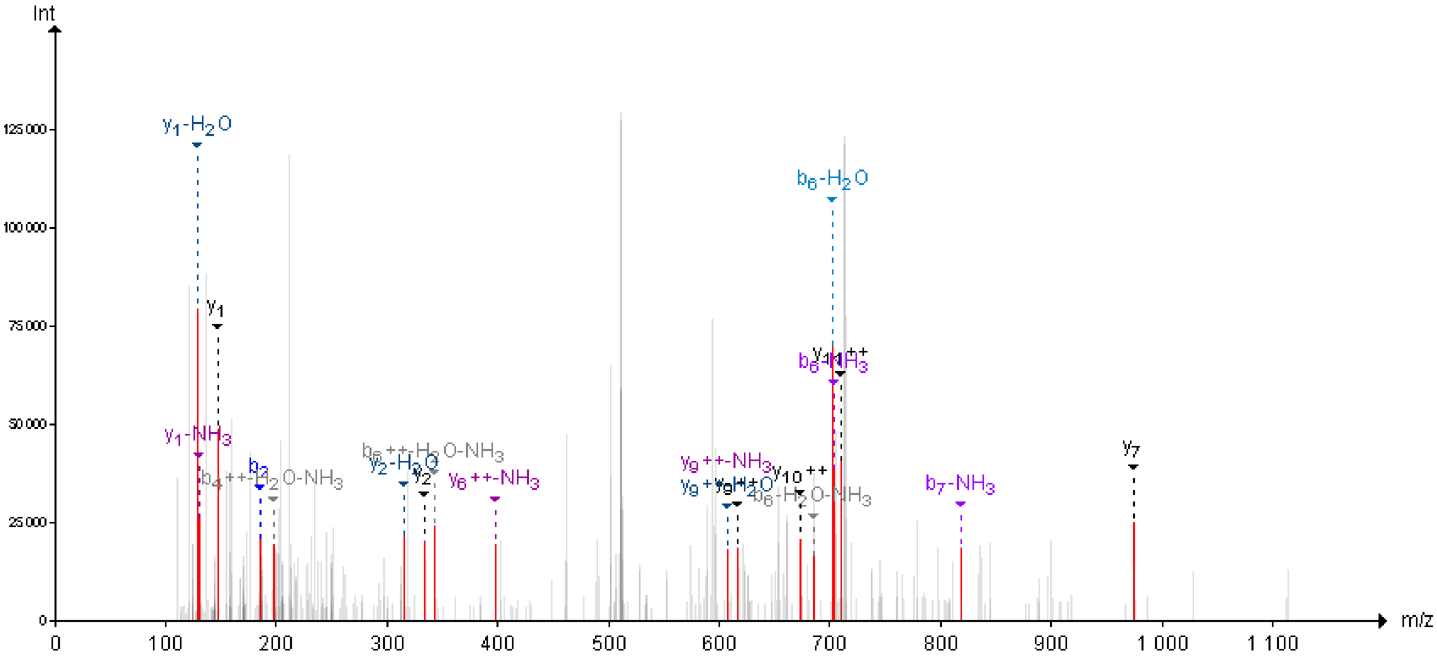

*AC:*

**Q58FG1**

*Protein existence:*

**PE5**

*Protein name:*

**Putative heat shock protein HSP 90-alpha A4**

*Gene name:*

**HSP90AA4P**

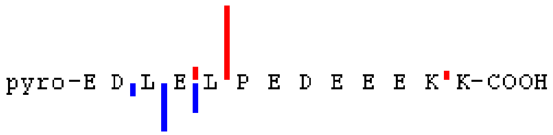


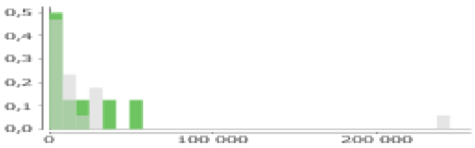


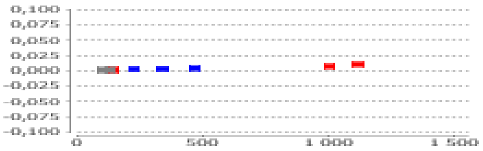


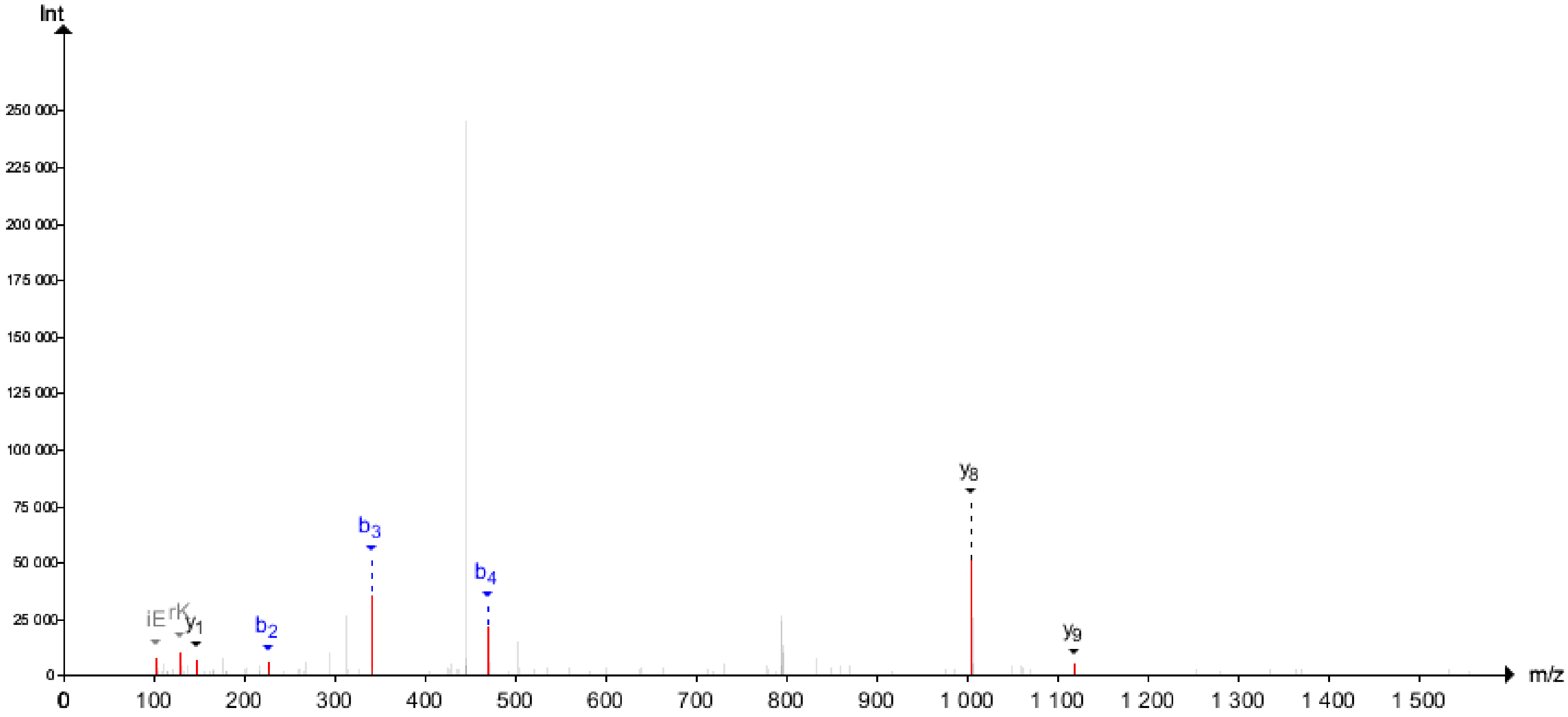

*AC:*

**Q9H853**

*Protein existence:*

**PE5**

*Protein name:*

**Putative tubulin-like protein alpha-4B**

*Gene name:*

**TUBA4B**

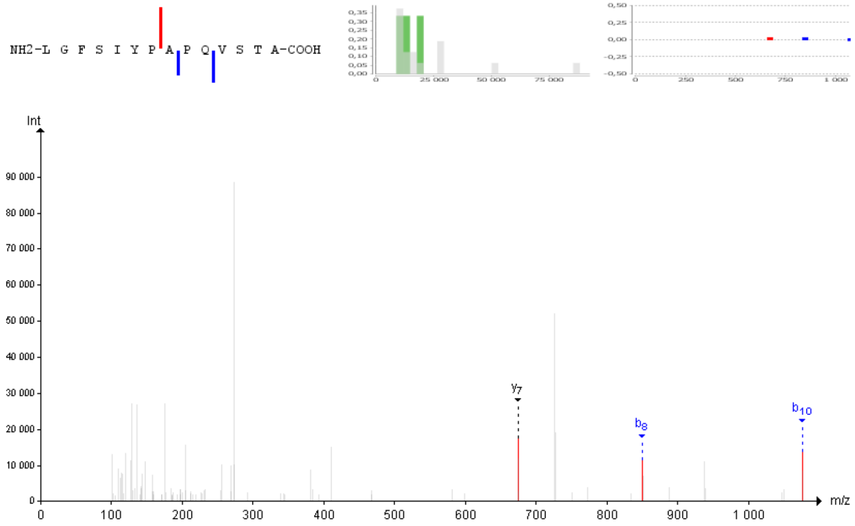

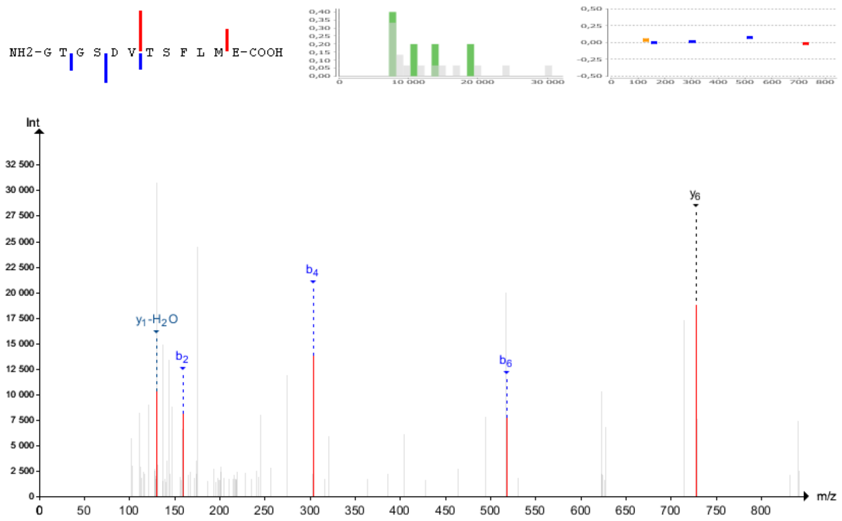

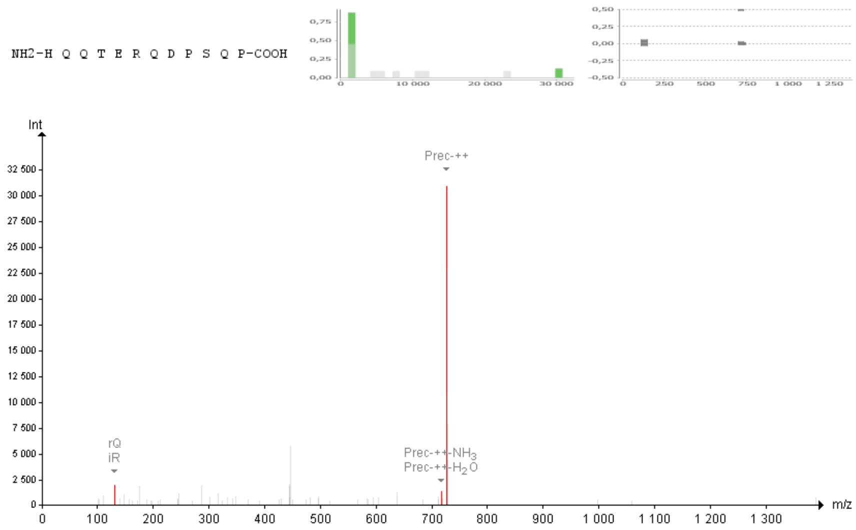

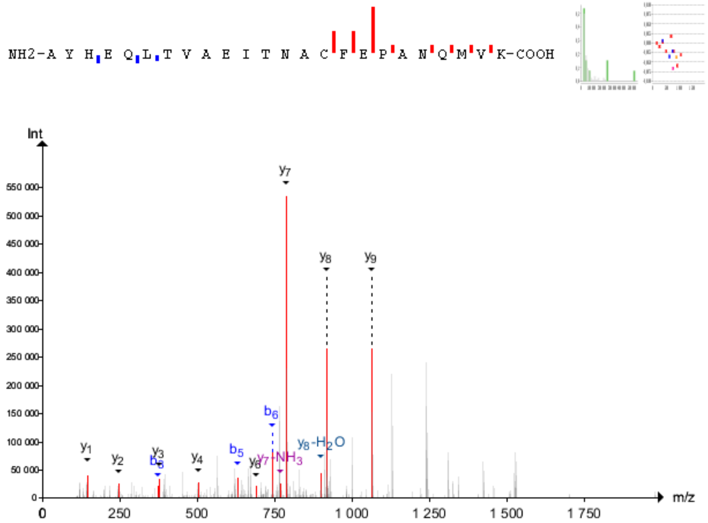

Supplement: Supplementary file 1 [file proteomes-08-00012-s001.zip › suppl/suppl_note1.pdf]
